# Decision theory approach to establishing an in-house histology laboratory in small hospitals

**DOI:** 10.1155/S1463924684000195

**Published:** 1984

**Authors:** Ik-Whan Kwon, Betty Yuen-Wah Yeung, Joe H. Kim, Jacqueline D. Frasca

**Affiliations:** 1 St. Louis University and Sister of St. Mary—Data Center St. Louis Missouri USA; 2 Lindell Hospital St. Louis Missouri USA; 3 Riders College Trenton New Jersey USA; 4 Medical Record Administration Cardinal Glennon Memorial Hospital for Children St. Louis Missouri USA; 5 School of Business Administration St. Louis University 3674 Lindell Blvd. St. Louis Missouri 63108 USA


Journal of Automatic Chemistry, Volume 6, Number 2 (April–June 1984), pages 91–93

### Decision theory approach to establishing an in-house histology laboratory in small hospitals

**Ik-Whan Kwon***

*St. Louis University and Sister of St. Mary—Data Center, St. Louis, Missouri, USA*

**Betty Yuen-Wah Yeung**

*Lindell Hospital, St. Louis, Missouri, USA*

**Joe H. Kim**

*Riders College, Trenton, New Jersey, USA*

**and Jacqueline D. Frasca**

*Medical Record Administration, Cardinal Glennon Memorial Hospital for Children, St. Louis, Missouri, USA*

Hospital laboratory directors have to decide whether to add an in-house histology laboratory to the clinical facilities or whether to subscribe the services of a nearby reference laboratory.

Selection is not an easy task. Many factors affect the choice: for example the present laboratory system, administration, pathology, engineering, medical records, medical staff etc. The choice becomes more difficult for small hospitals. The crux of the problem is that choices have to be made under conditions of uncertainty, and these uncertainties must first be methodically eliminated.

The Bayesian decision theory provides a strategy for making the best choice [1]. This theory is a tool that decision-makers can rely on in circumstances where several choices of action are available, but no exact information is available about the determinants of the outcome of those choices. In decision theory, choice is described as the interaction of groups or related constructs.

Modern decision theory is especially useful in situations where the results of the decision may depend upon the outcome of others. Most decision-making deals with more than one choice. Evidence of wide applicability of modern decision theory is apparent in such areas as business and economics [1–5] patient management [6 and 7], meteorology [8, 9 and 10], health care [11, 12 and 13] and laboratory tests [14 and 15].

Applying a systematic approach based on modern decision theory involves the following major steps:

(1) List all possible actions.
(2) List all possible outcomes.
(3) Assess the probability of each outcome from each action.
(4) Choose the best action based on likelihood and utility of the outcome.

Basically there are three choices available for providing histology services: (*a*) the complete services related to histology testing can be subscribed from a reference laboratory; (*b*) the services to be bought can be limited to processing and gross examination after which the hospital's pathologist will read the mounted slides; (*c*) an in-house histology department may be added to the clinical facilities with a full-time pathologist and a histo-technologist.

For the problem of choosing between these possibilities, decision theory can be an efficient and beneficial tool for laboratory directors. When the quantity of specimens to be tested is large and the cost of performing the analysis is reasonably low, an in-house histology department can seriously be considered. However, if the volume is rather small and the testing methods are numerous, the costs for an in-house facility and its staff may outweigh the possible advantages. Any choice under this circumstance, especially when a large initial capital investment is required, would be justified only after a careful feasibility study. All the variables pertinent to the alternatives must be incorporated into an integrated model in order to reach an optimum decision.

The objective of this research is to investigate how this common decision problem can be solved effectively. This approach can be used in other areas where similar decision environments exist. A theoretical model-building process is presented in the first half of this paper. The model is then applied to an actual case. Finally, the findings of the investigation are summarized in the conclusion.

### Model building process

In the most general form and under deterministic assumptions, the cost function can be expressed as:

$$Y_i = f(\bar{q}) \qquad (1)$$

which can be further expanded as:

$$Y_t = \alpha + \beta\bar{q} \qquad (2)$$

where $Y$ = total cost of testing histology specimens; $\alpha$ = fixed cost (for example instruments, salary and wages of pathologist/histo-technologist); $\beta$ = variable cost (for example stain, slides); $\bar{q}$ = expected number of histology specimens to be analysed; $t = i$ and $o$, where $i$ = histology specimens to be analysed at the in-house laboratory, and $o$ = to be analysed by a reference laboratory. At the break-even quantity of histology specimens, the laboratory director will be indifferent as to the selection of the method because the total cost for establishing an in-house

* *Correspondence to: School of Business Administration, St. Louis University, 3674 Lindell Blvd., St. Louis, Missouri 63108, USA.*

histology laboratory ($Y_1$) is equal to that of subscribing to a reference laboratory ($Y_o$), or

$$Y_1 = Y_o \quad (3)$$

Solving equation (3) for the break-even quantity ($q_k$),

$$\alpha_1 + \beta_1 \bar{q} = \alpha_o + \beta \bar{q}$$

$$\alpha_1 - \alpha_o = \beta_o \bar{q} - \beta_1 \bar{q} \quad (4)$$

$$q_k = \frac{\alpha_1 - \alpha_o}{\beta_o - \beta_1}$$

where $\alpha_1 > \alpha_o$ and $\beta_o > \beta_1$ and no economies of scale are assumed. [14].

Equation (4) indicates that if $\bar{q} > q_k$, then $Y_1 < Y_o$ (the in-house histology laboratory is preferred). On the other hand, if $\bar{q} < q_k$, then $Y_1 > Y_o$ (the use of a reference laboratory is preferred).

### Case

In 1981, a small, general, acute care hospital (about 95 beds) in the St. Louis Metropolitan Area decided to investigate the feasibility of adding a histology laboratory to its facilities. The hospital relied on a reference laboratory to test an average of 100 samples per month (i.e. 1200 surgical specimens annually).

To investigate and determine the feasibility, the Laboratory Director was asked to provide cost data for an in-house histology laboratory and for other possible alternatives. The Laboratory Director identified three possible choices and their related costs.

(1) The hospital would maintain the *status quo*, i.e. it would continue to send out all its histology specimens to a local reference laboratory at a cost of $13 per specimen. The services include gross-examination, processing, embedding and sectioning, mounting, staining and micro-examination.

(2) The specimens could be sent to the reference laboratory for the gross-examination and processing only for a fee of $3·25 per specimen. The hospital could then perform the microscopic examinations of the mounted slides using a part-time pathologist for an $18 000 annual salary.

(3) Add an in-house laboratory to the current facilities. Table 1 shows the costs of major capital equipment, salaries and reference laboratory costs. The capital equipment would cost the hospital $12 500; amortized over a period of five years, the annual cost of capital equipment would be approximately $2500 a year.

The wages and salaries of the pathologist and the histo-technologist were another major cost factor presented. Until the increased volume warranted a full-time staff, the existing volume could easily be handled by a part-time pathologist for an annual salary of $18 000.

In addition, a part-time histologist with an estimated annual wage of $7000 would be needed to process the tissue specimens on a day-to-day basis. Thus annual expenditures for wages/salaries would be approximately $25 000, making the annual total fixed cost of $27 500.

From table 1, the following coefficients for each parameter can be determined:

$\alpha_1 = \$2500 + \$25\,000$

$= \$2700$ per year

$\alpha_o = 0$

$\beta_1 = \$1{\cdot}00$

$\beta_o = \$13{\cdot}00.$

*Table 1. Cost information on an in-house histology department: a complete reference laboratory and a partial reference laboratory.*

| | | |
|---|---|---|
| Capital equipment | | $2500 per year |
| Automatic tissue processor | $4280·00 | |
| Tissue embedding center | 2241·00 | |
| Microtome/cryostat | 2236·00 | |
| Stainer | 2448·00 | |
| Water-bath | 306·00 | |
| File cabinet | 154·00 | |
| Blade | 210·00 | |
| Paraffin dispenser | 625·00 | |
| Total cost of capital equipment | $12500·00 | |
| Salary of part-time pathologist | | $18000 per year |
| Wages of part-time histo-technician | | $17000 per year |
| Cost for using a reference lab. for complete analysis | | $13 per specimen |
| Cost for using reference lab. for processing only | | 3·25 per specimen |
| Cost of slides and stains and other misc. | | $1·00 per specimen |

Accordingly, the cost functions become:

$Y_1 = 27\,500 + \bar{q}$ for complete in-house approach, and
$Y_o = 13\ \bar{q}$ for the complete send-out approach.

By equation (4), the break-even quantity ($q_k$) of surgical specimens required is:

$$q_k = \frac{\alpha_1 - \alpha_o}{\beta_o - \beta_1} = \frac{27\,500 - 0}{13 - 1} = 2292.$$

As the average number of specimens tested during the period of 1 April 1980–31 March 1981 (1200) is below the break-even quantity (2292), the present send-out approach is more cost-efficient.

To evaluate the second choice (processing and gross-examination by the reference laboratory, but other examinations by a part-time in-house staff), the following coefficients for each parameter can be determined:

$\alpha_1 = \$18\,000$
$\alpha_o = \$0$
$\beta_1 = \$3{\cdot}25$
$\beta_o = \$13.$

Accordingly, the cost functions become:

$Y_1 = 18\,000 + 3{\cdot}25\bar{q}$ for the partial in-house test approach;
$Y_o = 13\bar{q}$ for the complete send-out approach.

By equation (4), the break-even quantity of surgical specimens to be analysed is:

$$q_k = \frac{\alpha_1 - \alpha_o}{\beta_o - \beta_1} = \frac{18\,000 - 0}{13 - 3{\cdot}25} = 1846$$

As the quantity of samples required to justify this approach (1846) exceeds the average volume (i.e. 1200 per year), use of a part-time pathologist is not justified and consideration of this alternative should be eliminated.

### Limitation

Although it is evident that an in-house histology laboratory represents higher costs than the other possible choices, there are some advantages that cannot be based on monetary value.

Some of these advantages are: usefulness, especially in an emergency, when test results must be delivered to the physicians soon enough to be able to make a timely diagnosis and give the subsequent treatment. An in-house laboratory would increase speed, thus enhancing the service's usefulness.

An in-house laboratory would also increase the availability of slides for review by physicians. Furthermore, an in-house laboratory is consistent with the hospital's objectives of establishing complete clinical facilities.

Finally, an in-house laboratory can help to promote the prestige of hospitals, which in turn, may help to attract and keep dedicated professionals such as physicians, registered nurses etc.

### Conclusions

Decision theory can be employed efficiently and beneficially by hospital laboratory directors when they face a conflicting choice under the conditions of uncertainty.

Decision theory analysis was employed in this study to investigate the problem solution. Using only the monetary criteria, this analysis revealed that an addition of a histology department to the existing laboratory facilities is not cost effective. This study further revealed that a partial use of a reference laboratory is also not cost-justifiable. Accordingly, this research concluded that a use of the reference laboratory is the best course of action until such time that the volume of specimens exceeds the break-even volume (2292/year).

The Laboratory Director of the hospital recommended to the Administration not to change the current practice and to make continuous use of the reference laboratory. The administration accepted her recommendation for the time being.

### References

1. Kwon, I. W., *Statistical Decision Theory with Business and Economic Applications: A Bayesian Approach* (Van Nostrand: New York, 1978).
2. Bunn, D. W., *Journal of Marketing Research*, **16** (1979), 280–283.
3. Calabro, W., *Journal of Academy of Marketing Science*, **1** (1972), 12–24.
4. Green, P. E. and Harrison, P. J., *Operations Research Quarterly*, **24** (1973), 193–205.
5. Kwon, I. W. and Wei Wou, *Southern Business Review*, **4** (1978), 18–28.
6. Albert, D. A., *Millbank Memorial Fund Quarterly*, **56** (1978), 362.
7. Bataque, N. E. and Gorry, G. A., *Management Sciences*, **17** (1971), 421–434.
8. Julian, P. R. and Murphy, A. H., *Bulletin of American Meteorology Society*, **10** (1972), 957–965.
9. Kwon, I. W. and Doty, G. L., *Fifth Conference of Probability and Statistics in Atmospheric Science* (November 1977), 69–71.
10. Olsen, A. R., *Journal of Applied Meteorology*, **8** (1975), 970–973.
11. Kassire, J. P., *Seminar in Nuclear Medicine* (October 1978), 324–335.
12. Schoenbaum, S. C., McNeil, B. J., and Kavet, J., *New England Journal of Medicine*, **295** (1976), 759–765.
13. Stimson, D. H., *Management Sciences*, **16** (1969), 17–30.
14. Kwon, I. W. and Kim, J. H., *Journal of Chemical Education*, **58** (1981), 462–464.
15. Snyder, L., Levine, J., Stoy, R. and Conetta, A., *Analytical Chemistry*, **48** (1976), 942A–956A.

### NOTES FOR AUTHORS

*Journal of Automatic Chemistry* covers all aspects of automation and mechanization in analytical, clinical and industrial environments. The *Journal* publishes original research papers; short communications on innovations, techniques and instrumentation, or current research in progress; reports on recent commercial developments; and meeting reports, book reviews and information on forthcoming events. All research papers are refereed.

### Manuscripts

Two copies of articles should be submitted to the Editor. All articles should be typed in double spacing with ample margins, on one side of the paper only. The following items should be sent: (1) a title-page including a brief and informative title, avoiding the word 'new' and its synonyms; a full list of authors with their affiliations and full addresses; (2) an abstract of about 250 words—this should succinctly describe the scope of the contribution and highlight significant findings or innovations; it should be written in a style which can easily be translated into French and German; (3) the main text with sections and subsections numbered; (4) appendices (if any); (5) references; (6) tables, each table on a separate sheet and accompanied by a caption; (7) illustrations (diagrams, drawings and photographs) numbered in a single sequence from 1 upwards and with the author's name on the back of every illustration; captions to illustrations should be typed on a separate sheet. Papers are accepted for publication on condition that they have been submitted only to *Journal of Automatic Chemistry*.

### References

References should be indicated in the text by numbers following the author's name, i.e. Skeggs [6]. In the reference section they should be arranged thus:

to a journal

Manka, D. P., *Journal of Automatic Chemistry*, **3** (1981), 119.

to a book

Malmstadt, H. V., in *Topics in Automatic Chemistry*, Ed. Stockwell, P. B. and Foreman, J. K. (Horwood, Chichester, 1978), p. 68.

### Illustrations

Line diagrams are preferred to photographs. Original copies of diagrams and drawings should be supplied, and should be drawn to be suitable for reduction to the page or column width of the *Journal*, i.e. to 85 mm or 179 mm, with special attention to lettering size. Photographs may be sent as glossy prints or as negatives.

### Proofs and offprints

The principal or corresponding author will be sent galley proofs for checking and will receive 50 offprints free of charge. Additional offprints may be ordered on a form which accompanies the proofs.

*Manuscripts should be sent to the Editor: Dr Peter B. Stockwell, P.S. Analytical Ltd, 2 Eagles Drive, Tatsfield, Westerham, Kent TN16 2PB, UK.*

